# The genome sequence of the Autumnal Rustic, *Eugnorisma glareosa *(Esper, 1788)

**DOI:** 10.12688/wellcomeopenres.19987.1

**Published:** 2023-09-19

**Authors:** David C. Lees, Natural History Museum Genome Acquisition Lab, Darwin Tree of Life Barcoding collective, Wellcome Sanger Institute Tree of Life programme, Wellcome Sanger Institute Scientific Operations: DNA Pipelines collective, Tree of Life Core Informatics collective, Darwin Tree of Life Consortium

**Affiliations:** 1 Natural History Museum, London, England, UK

**Keywords:** Eugnorisma glareosa, Autumnal Rustic, genome sequence, chromosomal, Lepidoptera

## Abstract

We present a genome assembly from an individual male *Eugnorisma glareosa* (the Autumnal Rustic; Arthropoda; Insecta; Lepidoptera; Noctuidae). The genome sequence is 631.0 megabases in span. Most of the assembly is scaffolded into 30 chromosomal pseudomolecules, including the Z sex chromosome. The mitochondrial genome has also been assembled and is 15.39 kilobases in length. Gene annotation of this assembly on Ensembl identified 19,768 protein coding genes.

## Species taxonomy

Eukaryota; Metazoa; Eumetazoa; Bilateria; Protostomia; Ecdysozoa; Panarthropoda; Arthropoda; Mandibulata; Pancrustacea; Hexapoda; Insecta; Dicondylia; Pterygota; Neoptera; Endopterygota; Amphiesmenoptera; Lepidoptera; Glossata; Neolepidoptera; Heteroneura; Ditrysia; Obtectomera; Noctuoidea; Noctuidae; Noctuinae; *Eugnorisma*; *Eugnorisma glareosa*, (Esper, 1788) (NCBI:txid988114).

## Background

The Autumnal Rustic, *Eugnorisma glareosa*, is a medium-sized noctuid moth with a wingspan of about 32–38 mm. Its forewings usually exhibit dorsally crisp black markings on a light grey background, with two short black dashes or dots at the forewing base. The inner margins of the faintly outlined orbicular and reniform stigmata are in the form of conversely placed black arrow or axe heads. The former marking sometimes appears with adjacent black dots and often shows a diffuse rufous-grey or pinkish subterminal band. The forms in south-east England are more orange-brown and much darker forms are found in the Shetlands. As the vernacular name suggests, the adult moth emerges late in the Palaearctic Autumn, flying in the UK from August to October ( Randle *et al.*, 2019), and the moth overwinters as a small larva ( Waring *et al.*, 2017) *.*

The Autumnal Rustic has a preference for heathland, moorland or other types of open country such as downs and shingle beaches. The larva feeds on various low growing plants such as heathers and bedstraws as well as scrub birch and sallow ( Waring *et al.*, 2017). Genera fed on include *Calluna, Galium, Hieracium, Lactuca, Plantago, Poa, Rumex*, and *Salix* ( FUNET, 2023). The adult nectars on flowers such as heathers.

*E. glareosa* is generally fairly common and widespread in the western Palaearctic only, from northern and eastern Ireland (poorly represented in the west) to southern Scandinavia to Spain and the northern Mediterranean; avoiding Italy, but has relatively few records for eastern Europe ( GBIF Secretariat, 2023).

Populations in the UK have declined severely since 1970 ( Randle *et al.*, 2019); between 1968 and 2006 reductions in Rothamsted trap numbers of at least 94% were documented with an annual change decline of about 7% ( Conrad *et al.*, 2006).

Based on mitochondrial DNA, *E. glareosa* shows a single cluster on BOLD, BOLD:AAE3673 (21/08/2023) with southern Mediterranean exemplars slightly (up to 1.79%) divergent from the others; south-eastern English exemplars show identity with central European populations. The genus *Eugnorisma* Boursin, 1946 was partly revised by Varga *et al.* (1990) and these authors considered a previously suggested relative *Protexarnis* McDunnough, 1929 as morphologically convergent, and they also considered a possible relationship based on genital characteristics with *Eugraphe* Hübner,1821, *Paradiarsia* McDunnough, 1929, and *Xestia* Hübner, 1818. Surprisingly, the nearest neighbouring species of *E. glareosa* on BOLD is the dissimilar looking Double Dart *Graphiphora augur*, which is a mere 3.1% or more pairwise divergent in COI-5P (21/08/2023); other species of the genus *Eugnorisma*, including ones with similar wing patterns, are more distant. In this context the type species of the genus *Eugnorisma* is the Central Asian *Graphiphora insignata* Lederer 1853 (whereas *Graphiphora* Ochsenheimer, 1816 is currently considered monobasic, containing only *G. augur*). The genome sequence should therefore be useful in resolving the phylogenetic relationships of *Eugnorisma* using multiple loci. The data may also be useful to investigate genes that are potentially selected to influence forewing colouration in relation to darker resting substrates in northern Scotland and the Shetlands (see ( Kettlewell & Berry, 1969), and may also be relevant to the study of the evolution of melanism.

## Genome sequence report

**Figure 1.**
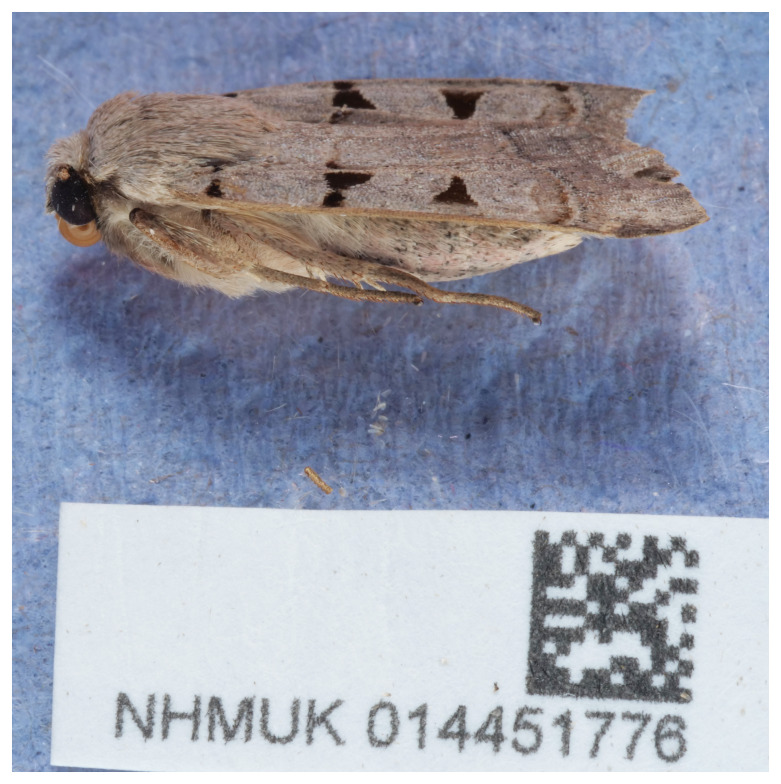
Photograph of the *Eugnorisma glareosa* (ilEugGlar6) specimen used for genome sequencing.

The genome was sequenced from one male *Eugnorisma glareosa* ( Figure 1) collected from Beinn Eighe (57.63, –5.35). A total of 42-fold coverage in Pacific Biosciences single-molecule HiFi long reads was generated. Primary assembly contigs were scaffolded with chromosome conformation Hi-C data. Manual assembly curation corrected 12 missing joins or mis-joins and removed 3 haplotypic duplications.

**Figure 2.**
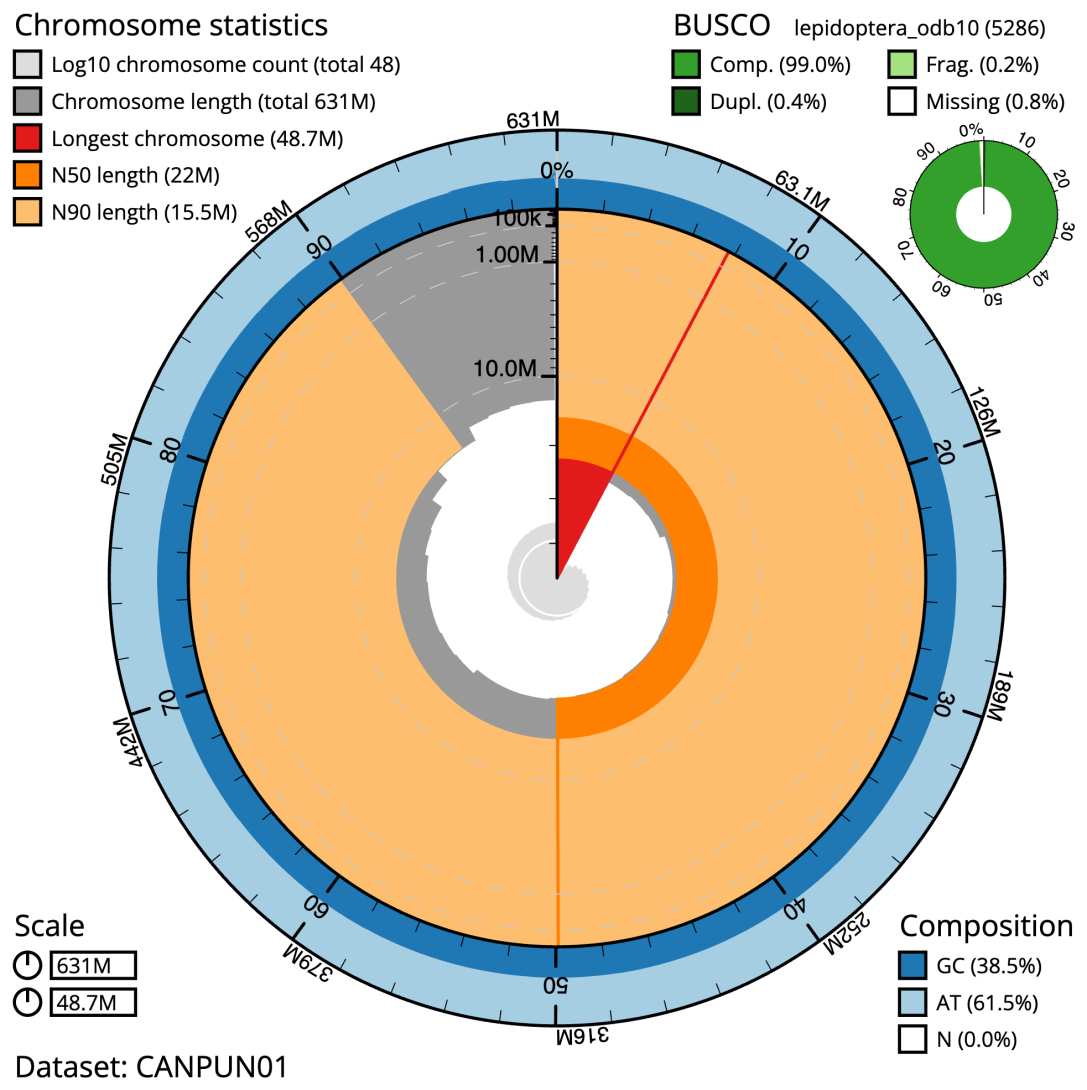
Genome assembly of *Eugnorisma glareosa*, ilEugGlar6.1: metrics. The BlobToolKit Snailplot shows N50 metrics and BUSCO gene completeness. The main plot is divided into 1,000 size-ordered bins around the circumference with each bin representing 0.1% of the 631,006,646 bp assembly. The distribution of scaffold lengths is shown in dark grey with the plot radius scaled to the longest scaffold present in the assembly (48,685,131 bp, shown in red). Orange and pale-orange arcs show the N50 and N90 scaffold lengths (21,954,397 and 15,478,121 bp), respectively. The pale grey spiral shows the cumulative scaffold count on a log scale with white scale lines showing successive orders of magnitude. The blue and pale-blue area around the outside of the plot shows the distribution of GC, AT and N percentages in the same bins as the inner plot. A summary of complete, fragmented, duplicated and missing BUSCO genes in the lepidoptera_odb10 set is shown in the top right. An interactive version of this figure is available at https://blobtoolkit.genomehubs.org/view/ilEugGlar6.1/dataset/CANPUN01/snail.

The final assembly has a total length of 631.0 Mb in 47 sequence scaffolds with a scaffold N50 of 22.0 Mb ( Table 1). Most (99.86%) of the assembly sequence was assigned to 30 chromosomal-level scaffolds, representing 29 autosomes and the Z sex chromosome. Chromosome-scale scaffolds confirmed by the Hi-C data are named in order of size ( Figure 2– Figure 5; Table 2). While not fully phased, the assembly deposited is of one haplotype. Contigs corresponding to the second haplotype have also been deposited. The mitochondrial genome was also assembled and can be found as a contig within the multifasta file of the genome submission.

**Table 1.** Genome data for *Eugnorisma glareosa*, ilEugGlar6.1.

Project accession data		
Assembly identifier	ilEugGlar6.1	
Species	*Eugnorisma glareosa*	
Specimen	ilEugGlar6	
NCBI taxonomy ID	988114	
BioProject	PRJEB57884	
BioSample ID	SAMEA14448290	
Isolate information	ilEugGlar6, male: thorax (DNA sequencing), head (Hi-C data)	
Assembly metrics *		*Benchmark*
Consensus quality (QV)	68	*≥ 50*
*k*-mer completeness	100%	*≥ 95%*
BUSCO **	C:99.0%[S:98.5%,D:0.4%], F:0.2%,M:0.8%,n:5,286	*C ≥ 95%*
Percentage of assembly mapped to chromosomes	99.86%	*≥ 95%*
Sex chromosomes	Z chromosome	*localised homologous pairs*
Organelles	Mitochondrial genome assembled	*complete single alleles*
Raw data accessions		
PacificBiosciences SEQUEL II	ERR10662013	
Hi-C Illumina	ERR10614863	
Genome assembly		
Assembly accession	GCA_947578425.1	
*Accession of alternate haplotype*	GCA_947579045.1	
Span (Mb)	631.0	
Number of contigs	106	
Contig N50 length (Mb)	12.1	
Number of scaffolds	47	
Scaffold N50 length (Mb)	22.0	
Longest scaffold (Mb)	48.7	
Genome annotation		
Number of protein-coding genes	19,768	
Number of gene transcripts	19,940	

* Assembly metric benchmarks are adapted from column VGP-2020 of “Table 1: Proposed standards and metrics for defining genome assembly quality” from ( Rhie *et al.*, 2021). ** BUSCO scores based on the lepidoptera_odb10 BUSCO set using v5.3.2. C = complete [S = single copy, D = duplicated], F = fragmented, M = missing, n = number of orthologues in comparison. A full set of BUSCO scores is available at https://blobtoolkit.genomehubs.org/view/ilEugGlar6.1/dataset/CANPUN01/busco.

**Figure 3.**
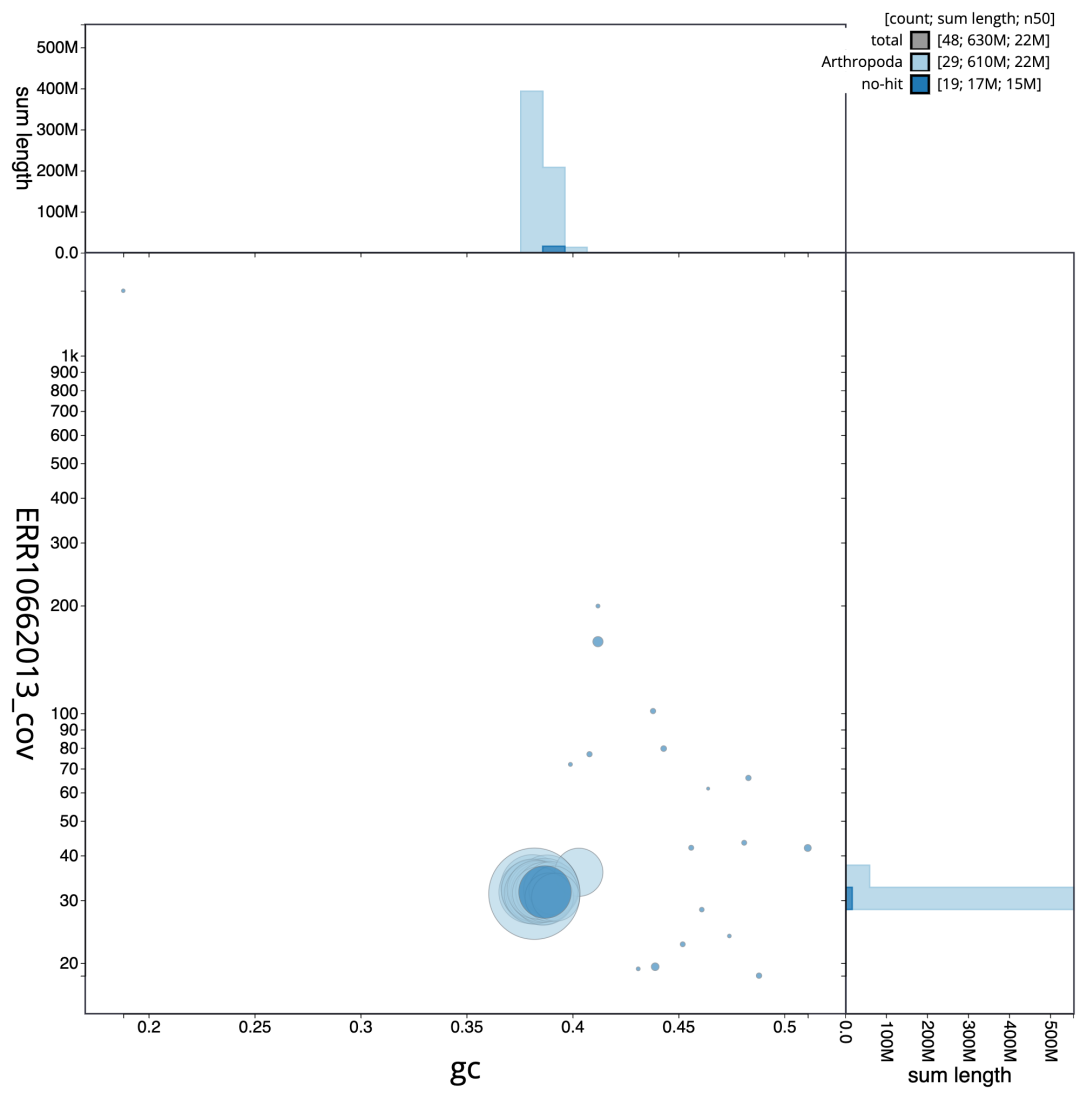
Genome assembly of *Eugnorisma glareosa*, ilEugGlar6.1: BlobToolKit GC-coverage plot. Scaffolds are coloured by phylum. Circles are sized in proportion to scaffold length. Histograms show the distribution of scaffold length sum along each axis. An interactive version of this figure is available at https://blobtoolkit.genomehubs.org/view/ilEugGlar6.1/dataset/CANPUN01/blob.

**Figure 4.**
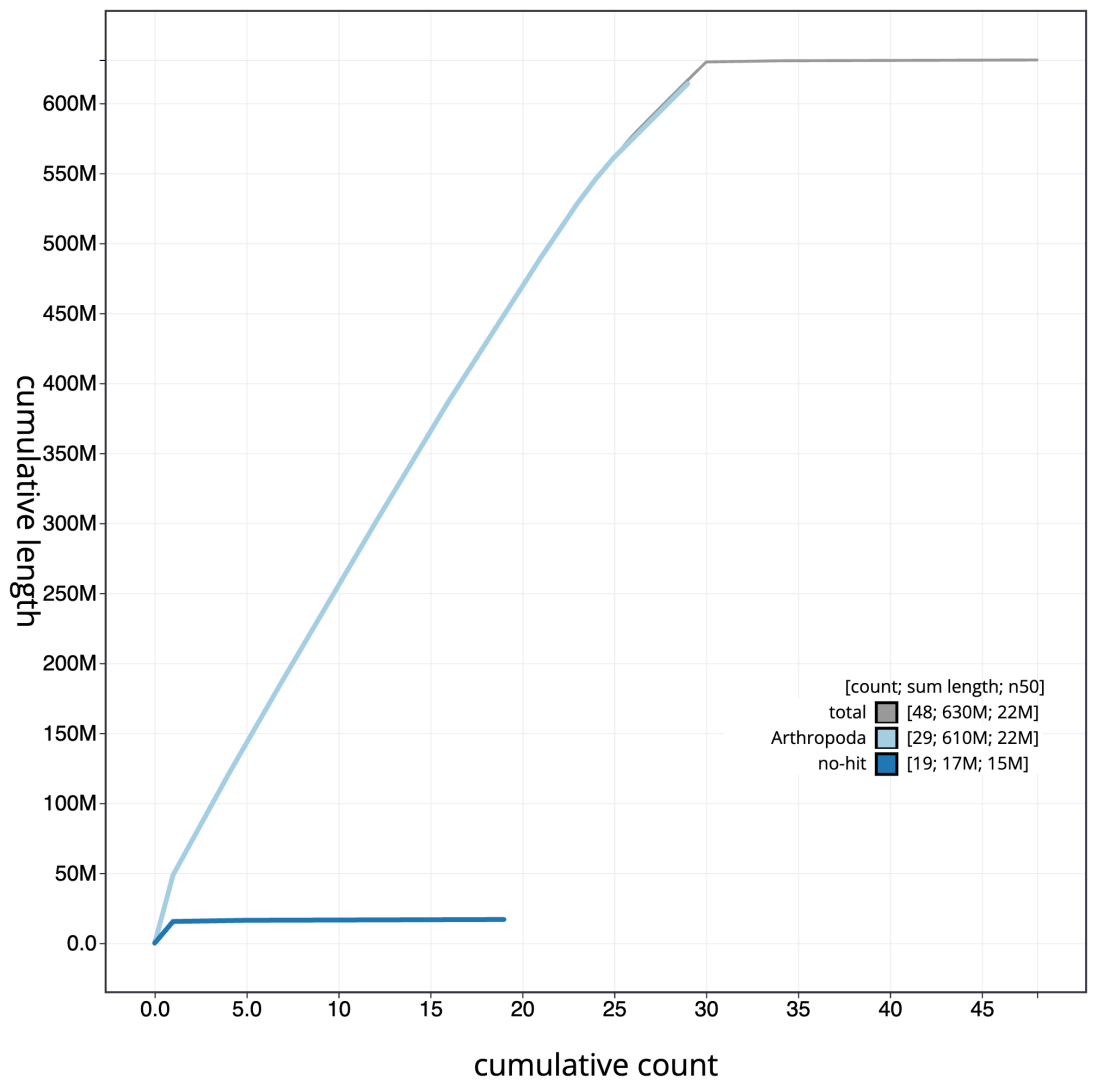
Genome assembly of *Eugnorisma glareosa*, ilEugGlar6.1: BlobToolKit cumulative sequence plot. The grey line shows cumulative length for all scaffolds. Coloured lines show cumulative lengths of scaffolds assigned to each phylum using the buscogenes taxrule. An interactive version of this figure is available at https://blobtoolkit.genomehubs.org/view/ilEugGlar6.1/dataset/CANPUN01/cumulative.

**Figure 5.**
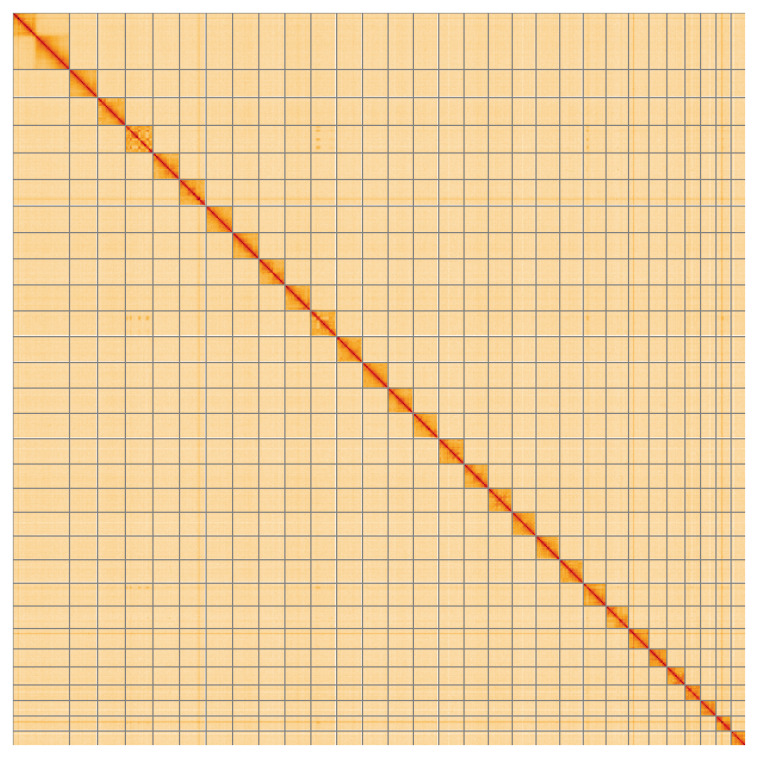
Genome assembly of *Eugnorisma glareosa*, ilEugGlar6.1: Hi-C contact map of the ilEugGlar6.1 assembly, visualised using HiGlass. Chromosomes are shown in order of size from left to right and top to bottom. An interactive version of this figure may be viewed at https://genome-note-higlass.tol.sanger.ac.uk/l/?d=EC1U7s0XTYaN5zpXpL2kUA.

**Table 2.** Chromosomal pseudomolecules in the genome assembly of *Eugnorisma glareosa*, ilEugGlar6.

INSDC accession	Chromosome	Length (Mb)	GC%
OX387958.1	1	24.06	38.5
OX387959.1	2	23.85	38.5
OX387960.1	3	23.73	38.5
OX387961.1	4	22.81	38.0
OX387962.1	5	22.79	38.0
OX387963.1	6	22.77	38.5
OX387964.1	7	22.51	38.5
OX387965.1	8	22.36	38.0
OX387966.1	9	22.35	38.5
OX387967.1	10	22.16	39.0
OX387968.1	11	22.15	38.5
OX387969.1	12	21.95	38.5
OX387970.1	13	21.77	38.0
OX387971.1	14	21.74	38.0
OX387972.1	15	21.72	38.5
OX387973.1	16	20.81	38.0
OX387974.1	17	20.58	38.5
OX387975.1	18	20.39	38.5
OX387976.1	19	20.37	38.5
OX387977.1	20	20.17	38.5
OX387978.1	21	19.57	38.5
OX387979.1	22	19.36	38.5
OX387980.1	23	17.45	39.0
OX387981.1	24	15.49	38.5
OX387982.1	25	15.48	38.5
OX387984.1	27	13.42	39.0
OX387985.1	28	13.2	39.0
OX387983.1	26	12.98	40.5
OX387986.1	29	12.92	39.0
OX387957.1	Z	48.69	38.0
OX387987.1	MT	0.02	19.0

The estimated Quality Value (QV) of the final assembly is 68 with *k*-mer completeness of 100%, and the assembly has a BUSCO v5.3.2 completeness of 99.0% (single = 98.5%, duplicated = 0.4%), using the lepidoptera_odb10reference set ( *n* = 5,286).

Metadata for specimens, spectral estimates, sequencing runs, contaminants and pre-curation assembly statistics can be found at https://links.tol.sanger.ac.uk/species/988114.

## Genome annotation report

The *Eugnorisma glareosa* genome assembly (GCA_947578425.1) was annotated using the Ensembl rapid annotation pipeline ( Table 1; https://rapid.ensembl.org/Eugnorisma_glareosa_GCA_947578425.1/Info/Index). The resulting annotation includes 19,940 transcribed mRNAs from 19,768 protein-coding genes.

## Methods

### Sample acquisition and nucleic acid extraction

A male *Eugnorisma glareosa* (specimen ID NHMUK014451776, individual ilEugGlar6) was collected from Beinn Eighe National Nature Reserve, Scotland, UK (latitude 57.63, longitude –5.35) on 2021-09-09, using a light trap. The specimen was collected and identified by David Lees (Natural History Museum) and dry frozen at –80°C.

DNA was extracted at the Tree of Life laboratory, Wellcome Sanger Institute (WSI). The ilEugGlar6 sample was weighed and dissected on dry ice with tissue set aside for Hi-C sequencing. Thorax tissue was disrupted using a Nippi Powermasher fitted with a BioMasher pestle. High molecular weight (HMW) DNA was extracted using the Qiagen MagAttract HMW DNA extraction kit. HMW DNA was sheared into an average fragment size of 12–20 kb in a Megaruptor 3 system with speed setting 30. Sheared DNA was purified by solid-phase reversible immobilisation using AMPure PB beads with a 1.8X ratio of beads to sample to remove the shorter fragments and concentrate the DNA sample. The concentration of the sheared and purified DNA was assessed using a Nanodrop spectrophotometer and Qubit Fluorometer and Qubit dsDNA High Sensitivity Assay kit. Fragment size distribution was evaluated by running the sample on the FemtoPulse system.

### Sequencing

Pacific Biosciences HiFi circular consensus DNA sequencing libraries were constructed according to the manufacturers’ instructions. DNA sequencing was performed by the Scientific Operations core at the WSI on a Pacific Biosciences SEQUEL II (HiFi) instruments. Hi-C data were also generated from head tissue of ilEugGlar6 using the Arima2 kit and sequenced on the Illumina NovaSeq 6000 instrument.

### Genome assembly, curation and evaluation

Assembly was carried out with Hifiasm ( Cheng *et al.*, 2021) and haplotypic duplication was identified and removed with purge_dups ( Guan *et al.*, 2020). The assembly was then scaffolded with Hi-C data ( Rao *et al.*, 2014) using YaHS ( Zhou *et al.*, 2023). The assembly was checked for contamination and corrected as described previously ( Howe *et al.*, 2021). Manual curation was performed using HiGlass ( Kerpedjiev *et al.*, 2018) and Pretext ( Harry, 2022). The mitochondrial genome was assembled using MitoHiFi ( Uliano-Silva *et al.*, 2023), which runs MitoFinder ( Allio *et al.*, 2020) or MITOS ( Bernt *et al.*, 2013) and uses these annotations to select the final mitochondrial contig and to ensure the general quality of the sequence.

A Hi-C map for the final assembly was produced using bwa-mem2 ( Vasimuddin *et al.*, 2019) in the Cooler file format ( Abdennur & Mirny, 2020). To assess the assembly metrics, the *k*-mer completeness and QV consensus quality values were calculated in Merqury ( Rhie *et al.*, 2020). This work was done using Nextflow ( Di Tommaso *et al.*, 2017) DSL2 pipelines “sanger-tol/readmapping” ( Surana *et al.*, 2023a) and “sanger-tol/genomenote” ( Surana *et al.*, 2023b). The genome was analysed within the BlobToolKit environment ( Challis *et al.*, 2020) and BUSCO scores ( Manni *et al.*, 2021; Simão *et al.*, 2015) were calculated.

Table 3 contains a list of relevant software tool versions and sources.

**Table 3.** Software tools: versions and sources.

Software tool	Version	Source
BlobToolKit	4.1.5	https://github.com/blobtoolkit/ blobtoolkit
BUSCO	5.3.2	https://gitlab.com/ezlab/busco
Hifiasm	0.16.1-r375	https://github.com/chhylp123/ hifiasm
HiGlass	1.11.6	https://github.com/higlass/higlass
Merqury	MerquryFK	https://github.com/thegenemyers/ MERQURY.FK
MitoHiFi	2	https://github.com/marcelauliano/ MitoHiFi
PretextView	0.2	https://github.com/wtsi-hpag/ PretextView
purge_dups	1.2.3	https://github.com/dfguan/purge_ dups
sanger-tol/ genomenote	v1.0	https://github.com/sanger-tol/ genomenote
sanger-tol/ readmapping	1.1.0	https://github.com/sanger-tol/ readmapping/tree/1.1.0
YaHS	1.2a	https://github.com/c-zhou/yahs

### Genome annotation

The BRAKER2 pipeline ( Brůna *et al.*, 2021) was used in the default protein mode to generate annotation for the *Eugnorisma glareosa* assembly (GCA_947578425.1) in Ensembl Rapid Release.

### Wellcome Sanger Institute – Legal and Governance

The materials that have contributed to this genome note have been supplied by a Darwin Tree of Life Partner. The submission of materials by a Darwin Tree of Life Partner is subject to the **‘Darwin Tree of Life Project Sampling Code of Practice’**, which can be found in full on the Darwin Tree of Life website here. By agreeing with and signing up to the Sampling Code of Practice, the Darwin Tree of Life Partner agrees they will meet the legal and ethical requirements and standards set out within this document in respect of all samples acquired for, and supplied to, the Darwin Tree of Life Project.

Further, the Wellcome Sanger Institute employs a process whereby due diligence is carried out proportionate to the nature of the materials themselves, and the circumstances under which they have been/are to be collected and provided for use. The purpose of this is to address and mitigate any potential legal and/or ethical implications of receipt and use of the materials as part of the research project, and to ensure that in doing so we align with best practice wherever possible. The overarching areas of consideration are:

•   Ethical review of provenance and sourcing of the material

•   Legality of collection, transfer and use (national and international) 

Each transfer of samples is further undertaken according to a Research Collaboration Agreement or Material Transfer Agreement entered into by the Darwin Tree of Life Partner, Genome Research Limited (operating as the Wellcome Sanger Institute), and in some circumstances other Darwin Tree of Life collaborators.

## Data Availability

European Nucleotide Archive: *Eugnorisma glareosa*. Accession number PRJEB57884; https://identifiers.org/ena.embl/PRJEB57884. ( Wellcome Sanger Institute, 2022) The genome sequence is released openly for reuse. The *Eugnorisma glareosa* genome sequencing initiative is part of the Darwin Tree of Life (DToL) project. All raw sequence data and the assembly have been deposited in INSDC databases. Raw data and assembly accession identifiers are reported in Table 1.

## Author information

Members of the Natural History Museum Genome Acquisition Lab are listed here: https://doi.org/10.5281/zenodo.4790042.

Members of the Darwin Tree of Life Barcoding collective are listed here: https://doi.org/10.5281/zenodo.4893703.

Members of the Wellcome Sanger Institute Tree of Life programme are listed here: https://doi.org/10.5281/zenodo.4783585.

Members of Wellcome Sanger Institute Scientific Operations: DNA Pipelines collective are listed here: https://doi.org/10.5281/zenodo.4790455.

Members of the Tree of Life Core Informatics collective are listed here: https://doi.org/10.5281/zenodo.5013541.

Members of the Darwin Tree of Life Consortium are listed here: https://doi.org/10.5281/zenodo.4783558.
