# Supplementary material for: The genome sequence of the Autumnal Rustic, Eugnorisma glareosa (Esper, 1788)
Source: Wellcome Open Res. 2023 Sep 19;8:410. [Version 1] doi: 10.12688/wellcomeopenres.19987.1 (PMC11109686; doi:10.12688/wellcomeopenres.19987.1)
Supplement: Peer review history

# Reviewer report: Reviewer response for version 1

**DOI:** 10.21956/wellcomeopenres.22133.r77742

**Published:** 2024-05-13

**Authors:** Andrea Battisti

**Affiliations:** 1 University of Padova, Padova, Italy

I do not have major concerns about the manuscript, only a few comments in the attached PDF file which can be viewed here.

 I congratulate the authors for the quality of the work that will contribute to the knowledge on the Lepidoptera genomic science and make it possible to compare the species with the few others for which a full genome is available.

Are sufficient details of methods and materials provided to allow replication by others?

No

Is the rationale for creating the dataset(s) clearly described?

Yes

Are the datasets clearly presented in a useable and accessible format?

Yes

Are the protocols appropriate and is the work technically sound?

Yes

Reviewer Expertise:

entomology

I confirm that I have read this submission and believe that I have an appropriate level of expertise to confirm that it is of an acceptable scientific standard.

# Reviewer report: Reviewer response for version 1

**DOI:** 10.21956/wellcomeopenres.22133.r80348

**Published:** 2024-04-29

**Authors:** Fahad Alqahtani

**Affiliations:** 1 King Abdulaziz City for Science and Technology, Riyadh, Saudi Arabia

In "The genome sequence of the Autumnal Rustic, *Eugnorisma glareosa* (Esper, 1788)," the authors have achieved a highly complete genome assembly at the chromosome level for a male Autumnal Rustic, *Eugnorisma glareosa* (Esper, 1788).

 The genome assembly was reconstructed using two sequencing technologies: Pacific Biosciences SEQUEL II and Hi-C Illumina. The completeness of the genome assembly was assessed using BUSCO analysis, which indicated a genome size of 631 megabases, with 99% of common genes in the lepidoptera_odb10 present.

 There is just one minor comment that should be addressed: 'Pretext' in the Genome Assembly section should be corrected to 'PretextView' to match the name in Table 3.

Are sufficient details of methods and materials provided to allow replication by others?

Yes

Is the rationale for creating the dataset(s) clearly described?

Yes

Are the datasets clearly presented in a useable and accessible format?

Yes

Are the protocols appropriate and is the work technically sound?

Yes

Reviewer Expertise:

Bioinformatics

I confirm that I have read this submission and believe that I have an appropriate level of expertise to confirm that it is of an acceptable scientific standard.
